# Porphyrin-based electrospun nanomaterials for life science applications

**DOI:** 10.1016/j.bbadva.2025.100160

**Published:** 2025-04-16

**Authors:** Ana C. Mendes, Nuno M.M. Moura, M. Amparo F․ Faustino, M. Graça P.M.S. Neves, Ioannis S. Chronakis

**Affiliations:** a Research Group for Food Production Engineering, Laboratory of Nano-Bioscience, DTU-Food, Technical University of Denmark, Henrik Dams Allé B202, 2800 Kgs. Lyngby, Denmark; b LAQV-RQUIMTE, Department of Chemistry, University of Aveiro, Campus Universitário de Santiago, 3810-193 Aveiro, Portugal

**Keywords:** Nano-microfibers, Nano-microtechnology, Encapsulation, Porphyrins, Photodynamic therapy, Photodynamic-based applications, Drug delivery, Sensors

## Abstract

Porphyrins are renowned for their utility in photocatalysis, electronics, sensors, solar cell dyes, and environmental remediation. However, their potential in life sciences applications remains underexplored, particularly in the context of encapsulation via electrospinning. This review examines recent advancements (2000–2025) in electrospinning techniques for encapsulating porphyrins, highlighting their unique properties, bioactivities, and versatile applications in life sciences. By combining the strengths of porphyrins and electrospinning technology, researchers can unlock transformative solutions for various life-science challenges, which include photodynamic therapy (PDT), composite materials for anti-microbial applications, sensors, and drug delivery. These efforts could push porphyrin-encapsulated materials from research concepts to applied societal solutions, addressing critical needs in healthcare and beyond to other fields.

## Highlights

• Porphyrins have great potential in life sciences via electrospinning (ES).
• Research advancements from 2000 to 2025 of ES of porphyrins are disclosed.
• ES boosts porphyrin applications in photodynamic therapy, sensors, and drug delivery.

## 1 Introduction

Porphyrins and their analogs have been receiving increased attention due to their pivotal role in biological processes, and unique (photo)physicochemical properties such as strong light absorption, fluorescence, and redox catalytic activity. These features make them invaluable in several chemical, environmental, electronics, and medical applications [[1], [2], [3]]. Several studies have shown that the functional features of porphyrins can be boosted by immobilizing or encapsulating them on polymeric substrates [4,5].

In recent years, electrospinning has gained attention as an innovative method for encapsulating (bio)active compounds and cells within nano-micro fiber-shaped structures, due to their high surface area per unit mass, high porosity, and tunable functional properties [6,7].

Despite porphyrin’s well-documented utility in fields of photocatalysis, electronics, sensors, solar cell dyes, and environmental remediation [8,9], the potential of porphyrins in life-science applications when encapsulated via electrospinning remains less explored. This review highlights recent research advancements (2000–2025) in using electrospinning techniques to encapsulate porphyrins, showcasing their untapped potential in life sciences and related applications.

## 2 Porphyrins properties, production, bioactivities, and applications

**Fig. 1:**
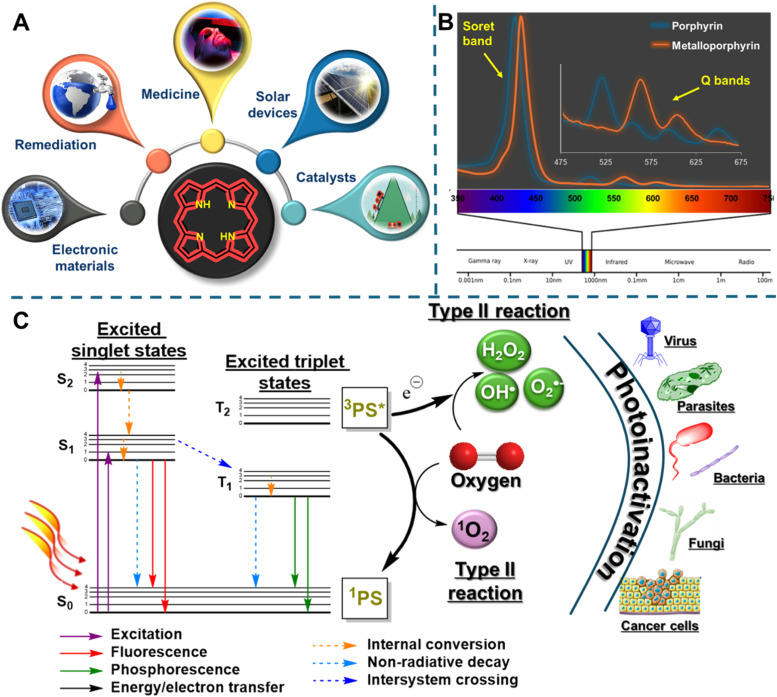
A) Porphyrin macrocycle and representative applications, B) characteristic absorption spectra of free-base 5,10,15,20-tetraarylporphyrins (blue line) and their metalloporphyrins counterparts (orange line), C) simplified Jablonski diagram and ROS mechanisms involved in PDT approaches (For interpretation of the references to color in this figure legend, the reader is referred to the web version of this article.).

Porphyrins and their analogs are highly conjugated aromatic macrocycles consisting of four pyrrolic type units linked by methinic bridges (−CH=) (Fig. 1A). Beyond their well-established roles in essential biological processes such as electron transfer, photosynthesis, and respiration, they are increasingly valued for their unique physicochemical properties, including strong light absorption in the visible region of the electromagnetic spectrum, fluorescence emission, and the ability to participate as a catalyst in several redox reactions, which enable a wide range of applications. The potential of natural and synthetic porphyrins is well recognized, particularly in the development of new therapeutic strategies for medicine, (photo)catalytical applications, the design of electronic devices, sensors, as dyes for DSSC cells, and environmental remediation, among others (Fig. 1A) [1,2].

Some applications of these highly colored macrocycles are related to their unique absorption features, which include a highly intense absorption (Soret band) at approximately 400 nm and four less intense Q-bands between 500–700 nm. These absorption bands correspond to π-π* electronic transitions from the ground state (S_0_) to the second (S_2_) and first (S_1_) excited states, respectively (Fig. 1B). The relative intensities of these bands provide valuable information regarding the type and position of substituents on the porphyrinic core and its oxidation state [10,11].

Another important feature of these macrocycles is their ability to coordinate with a wide range of metal ions, which significantly changes their UV-Vis spectrum, namely red-shifts in the Soret band and a reduction in the number of Q-bands, due to increased symmetry, which result in one or two Q-bands between 500–630 nm. Such spectral modifications are particularly relevant in detecting and controlling metal ions in sensing and adsorption processes. Porphyrins and metalloporphyrins can also exhibit strong fluorescence, typically with emission bands centered between 650 and 750 nm [11]. Although this feature is strongly influenced by substituents, metal ion, solvent, and other experimental conditions like pH, it is particularly appealing for sensing, fluorescence-guided surgery, and tumor visualization applications [1,3,11].

One of the most significant features of porphyrins for therapeutic, such as photodynamic therapy (PDT), antimicrobial photodynamic therapy (aPDT), and photocatalytic applications, is their ability to generate reactive oxygen species (ROS) in the presence of dioxygen (^3^O_₂_) upon activation with visible light. When these porphyrinic photosensitizers (PS) are irradiated with visible light, they are promoted to an excited singlet state (S_n_; *n* = 1–2), which can return to the ground state (S_0_) either through non-radiative processes (internal conversion) or by emitting fluorescence. Alternatively, their short-lived excited singlet state (S_1_) can undergo intersystem crossing (ISC) to form an excited triplet state (T_1_). The long-lived T_1_ can then generate cytotoxic ROS through type I (electron transfer, e.g., O_2_^●–^, OH^●^, H_2_O_2_) and/or type II (energy transfer, ^1^O_2_) reactions (Fig. 1C) [[12], [13], [14]]. These ROS can interact with biological components, leading to cell death and consequently damage cancer cells, eradicate pathogenic microorganisms (*e.g.* bacteria, fungi, viruses), and oxidize dyes or other relevant pollutants.

In recent years, special attention has been devoted to immobilizing porphyrin derivatives on various solid supports or matrices, unlocking new practical perspectives in several of the mentioned applications. For instance, immobilization facilitates the recovery and reuse of these materials, making processes such as photocatalysis, sensing, aPDT, and pollutant adsorption more cost-effective and environmentally sustainable by reducing leaching or contamination risks. Among the different approaches to incorporating porphyrin and analogs into solid matrixes, electrospinning techniques have gained particular attention [15]. The integration of porphyrins into electrospun fibers produces new advanced materials with enhanced functionalities, such as improved light-harvesting efficiencies for solar energy applications, high-performance sensors for detecting environmental pollutants, antimicrobial and self-sterilizing coatings, and drug delivery systems, among others [[16], [17], [18], [19], [20]]. Some of these applications are highlighted in the section below.

## 3 Encapsulation of porphyrins using electrospinning

**Fig. 2:**
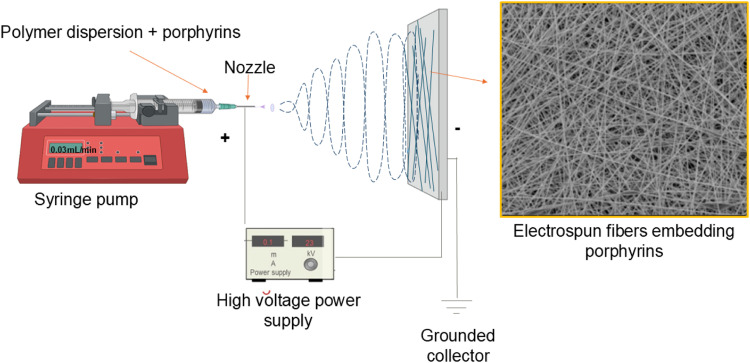
Schematics of Electrospinning setup (For interpretation of the references to color in this figure legend, the reader is referred to the web version of this article.).

Electrospinning has been widely used for the encapsulation of sensitive compounds, and suiting a broad range of applications [6,7]. These processes utilize high-voltage electrostatic fields to charge the surface of (bio)polymer solutions, leading to the ejection of a liquid jet from a spinneret (nozzle) towards the collector of opposite charge (Fig. 2). When the electric field exceeds the surface tension, the jet forms electrospun fibers, enabling efficient encapsulation [6,7].

**Fig. 3:**
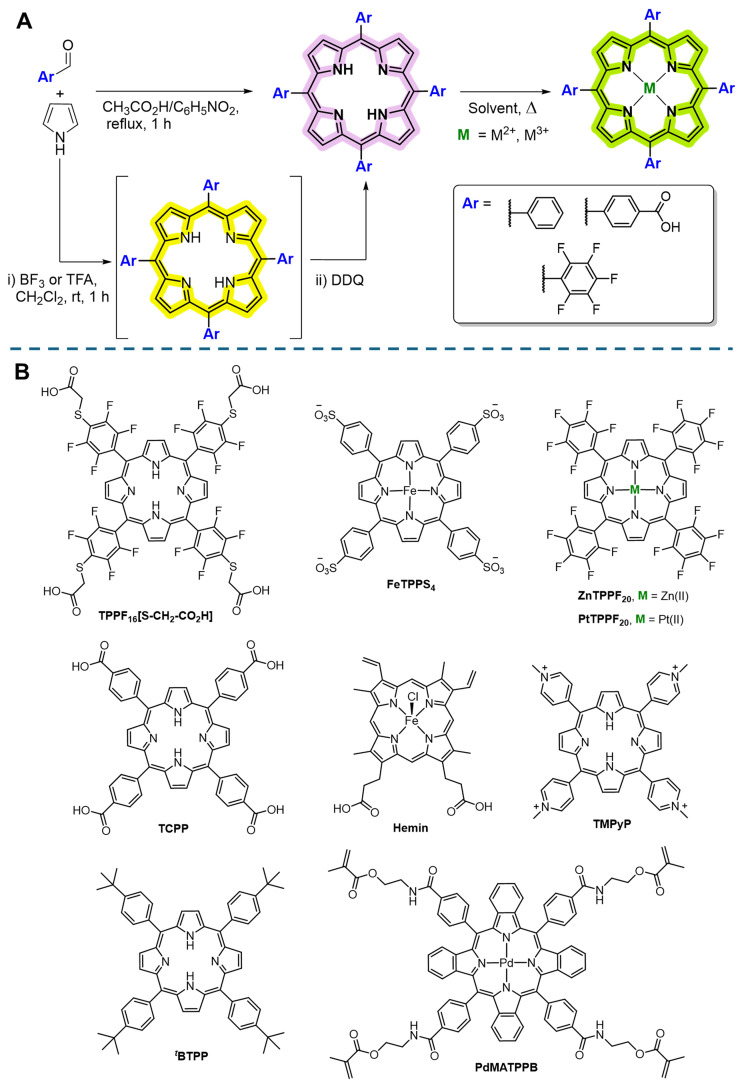
A) Main synthetic approaches used to prepare free-base *meso*-tetraarylporphyrins and their metal complexes and B) structures of porphyrinic derivatives encapsulated within electrospun fibers (For interpretation of the references to color in this figure legend, the reader is referred to the web version of this article.).

With respect to the encapsulation of porphyrins within electrospun nanofibers, the literature survey shows that, with few exceptions, most studies selected symmetric 5,10,15,20-tetraarylporphyrins as free-bases or coordinated with metal ions to be incorporated into nanofibers. These synthetic porphyrins are considered an excellent alternative to natural porphyrins due to their easy synthetic accessibility and straightforward structures. Synthetic access to these types of porphyrins typically involves the condensation of pyrrole with suitable aldehydes. This is generally carried out under: i) acidic conditions (propionic or acetic acid) under reflux and oxidative conditions; or ii) in CH₂Cl₂ at room temperature in dioxygen and light absence, using trifluoroacetic acid (TFA) or BF₃ as catalysts, to maximize the formation of the porphyrinogen intermediate, followed by an oxidative step with DDQ or *p*-chloranil (Fig. 3A). This two-step approach is preferred for aldehydes that are unstable under refluxing acidic conditions [21]. Other useful synthetic approaches include microwave-mediated reactions both under classical acidic conditions [22] or using other catalytic agents, such as iodine, to promote the porphyrin macrocycle formation [23].

Several types of porphyrins have been encapsulated within electrospun fibers for different life sciences applications; some of the structures referred to in the following topics are shown in Fig. 3B.

### 3.1 Drug delivery for photodynamic therapy (PDT)

Functional electrospun nanofibers, with their high surface-area-to-volume ratio, drug-loading capacity, controlled delivery, biocompatibility, and biodegradability, enhance PDT for oncological and infectious diseases by enabling localized treatment with higher efficacy and reduced toxicity compared to systemic drug administration. However, there are very limited studies exploring the localized delivery, effect, and cytotoxicity of photosensitizers (PS) in tumor sites [24].

The photodynamic effect of porphyrins depends on their purity, chemical stability, water solubility, ^1^O_2_ quantum yield (Φ_Δ_). Furthermore, strong absorption in the near-infrared (NIR) region (650–850 nm) is required for deeper tissue penetration, as light scattering is reduced in this “phototherapeutic window”. Besides, the subcellular localization of porphyrins in different organelles (mitochondria, lysosomes, endoplasmic reticulum, etc.) is essential to direct the cell death mechanism [25]. The photodynamic effect is feasible due to the very short lifetime of ^1^O_2_ (around 3.6 µs in water) and short action radius (0.01–0.55 µm) near the site of photosensitization in the illuminated area [26]

Core-shell electrospun nanofiber, using of poly(vinyl alcohol) (PVA) and gelatin (Gel), in the shell, and a TPPF_16_[S–CH_2_–COOH] porphyrin in the core, have also been tested as localized delivery systems of PSs for the treatment of cervical cancer [18]. An initial fast-release stage of the TPPF_16_[S–CH_2_–COOH] from the fibers, followed by a continuous release for at least 9 days was observed. The nanofibers were found to selectively inhibit the proliferation of cancer cells under light irradiation when compared to the dark, and a higher phototoxic effect against tumor cells compared with non-tumor cells was observed. Depending on the combination of the matrix polymers and nanofiber structure, core–shell nanofibers can provide a more sustained and controlled release than single ones, since the shell functions as a barrier and the core can act as the carrier of the drug molecules. The utilization of biodegradable polymers will also avoid surgical removal of the localized drug delivery systems at the end of their therapeutic lifetime, particularly for the cervical cancer treatment [18,27]. Thus, electrospun nanofibers can be effectively utilized for the localized delivery of PS on tumor cells for a longer time, as well as maintaining therapeutically effective drug(s) concentrations in the target tissue [18,28]. It is also to note that cell–material interaction can occur due to the nanofiber’s structure, which is similar to the extracellular matrix (ECM) [4]. In addition, phototherapeutic nanofibers can also function as theragnostic nanoplatforms, enabling simultaneous diagnosis and therapy [29].

Other studies have been shown the potential of electrospinning in PDT [5], demonstrating that electrospun fibers loaded with PS offer inhibitory effects against bacterial, fungal, and cellular growth (including cancerous cells) under low light doses and PS concentrations. The most commonly used polymers are polyurethane and polystyrene, while 5,10,15,20-tetraphenylporphyrin (TPP) and various phthalocyanine derivatives are the most studied PS. Overall, the interaction between the PS and the polymer matrix, as well as its arrangement within the fibers, is crucial for achieving effective results. Additionally, the average fiber diameter plays a key role in O_2_ diffusion and photodynamic activity. Most materials exhibit photoactive effects only on the surface, without PS diffusion from the fibers. Despite promising outcomes, only a limited range of PS and polymers have been explored, which highlights the need for further investigation and is expected to guide future studies involving new PS drugs, as electrospun scaffolds hold significant potential, especially in wound healing applications [5].

### 3.2 Antibacterial nanofibers

Electrospun nanofibers loaded with antimicrobial PS can be used for the inactivation of resistant bacteria strains. Typically, for antimicrobial photodynamic applications, cationic PSs are more effective as they bind adequately to the negatively charged bacteria surface, particularly in Gram-negative bacteria. In a recent study ethylcellulose (EC) nanofibers loaded with [5,10,15,20-tetrakis(pentafluorophenyl)porphyrinato]zinc(II) (TPPF_20_-Zn) was found to generate ROS capable to photo-kill *Escherichia coli*, in planktonic suspension [19]. Several other studies also evaluated the use and efficiency of both *in vitro* and *in vivo* models of electrospun nanofibers with PS to induce light-induced antibacterial activity against a range of bacteria (such as *S. aureus, S. aureus* subsp. aureus, *Staphylococcus saprophyticus* subsp. bovis, Methicillin-resistant *S. aureus* (MRSA), *E. coli, E. coli* DH5 alpha and *E. coli* K-12 and *Bacillus subtilis*, and Meropenem-resistant *Pseudomonas aeruginosa* (MRPA), among others), as discussed in a recent review [24].

PCN-224, a type of porous coordination network (PCN) embedded with a 5,10,15,20-tetrakis(4-carboxyphenyl)porphyrin (TCPP), has gained significant attention due to its exceptional properties for PDT applications in cancer, including its versatility, controllable synthesis, and remarkable stability in both acidic and basic environments. Furthermore, PCN-224 was shown to exhibit strong bactericidal activity when exposed to visible light, offering new possibilities for developing advanced, light-activated self-disinfecting materials [30]. Thus, a photosensitive metal-organic framework (MOF) material, PCN-224 nanoparticles (NPs), was synthesized via a solvothermal method and successfully incorporated into electrospun polyacrylonitrile (PAN) nanofibers. The PAN prevented drug leakage and provided a flexible support material for the PCN-224 NPs. aPDT experiments demonstrated that the PAN-PCN nanofiber membrane effectively eliminated *E. coli* and *S. aureus* at low PCN-224 concentrations (0.6 wt%), with bacterial reduction of 3.00 and 4.70 log of % survival, respectively, under illumination. The antibacterial effect was attributed to the strong oxidation of bacterial targets, mediated by the ^1^O_2_ generated. Despite their strong bactericidal properties, the nanofiber membranes PAN-PCN exhibited good biocompatibility, as shown by MTT assays (cell survival rates above 85 % at the highest tested concentration) [30].

This research offers a promising solution to combat the rising issues of drug-resistant pathogens by using phototoxic composite nanofiber membranes with photodynamic effects, which could be applied to prevent the spread of infections [30]. Overall, electrospun photoactive fibers display excellent photostability and can be used for the development of antimicrobial skin patches for dermal applications or self-sterilizing surfaces and other photodynamic biomedical applications.

Hemin, a natural porphyrin, known for its remarkable biological properties and its ability to create composite materials, has been used in several biomedical applications [31]. Thus, the development of biocompatible composites based on electrospun poly(3-hydroxybutyrate) (PHB) and hemin was conducted, showing that hemin enhances the functionality of PHB fibers by improving their mechanical strength, antimicrobial activity, and structural properties. The incorporation of 1–5 wt% hemin significantly impacted the supramolecular structure and morphology of the electrospun fibers, which had an impact on the crystallinity of the fiber composites [31,32]. The addition of hemin increased elongation at break by 1.5 times and tensile strength by 3 times, while eliminating surface defects at higher concentrations. Moreover, the antimicrobial properties of the PHB-Hemin composites against *E. coli* and *S. aureus* were confirmed. The stability [33] and enhanced properties of the developed fiber composites, make them promising for biomedical applications, including wound-healing bandages and combined drug formulations [31].

Nanofibrous of electrospun polyanionic poly(γ-glutamic acid) (γ-PGA) with the cationic PS 5,10,15,20-tetrakis(1-methylpyridinium-4-yl)porphyrin tetra-tosylate (TMPyP), stabilized through chemical crosslinking were also developed [34]. The hydrophilic γ-PGA provided a moist environment conducive to wound healing, while TMPyP facilitated potent antibacterial activity through the controlled release of cytotoxic ROS at low porphyrin concentrations (e.g., 0.1 wt%). These electrospun materials demonstrated a broad-spectrum antibacterial efficacy, biocompatibility, and minimal toxicity to L929 murine fibroblasts, and red blood cells. *In vivo* studies confirmed the efficacy of those materials to reduce inflammation and promote faster wound healing with negligible local side effects, highlighting their potential as an effective infection-resistant wound dressing.

### 3.3 Drug metabolism

New generation biomimetic catalysts using electrospun nanofibers and metalloporphyrin for *in vitro* mimicking of hepatic drug metabolism can find innovative applications in early-stage drug discovery [35]. Indeed, synthetic metalloporphyrins show structural similarity to the heme-type prosthetic group of cytochrome P450, a primary hepatic enzyme involved in oxidative drug biotransformation and can be used to evaluate the catalytic processes involved in these oxidations. In line with this, composites based on amino-functionalized magnetic nanoparticles (MNP) electrostatically bound to [5,10,15,20-tetrakis(4-sulfophenyl)porphyrinato]iron (FeTPPS_4_) and subsequently immobilized within electrospun polylactic acid (PLA) nanofibers were developed. The biomimetic efficiency of these nanostructures was investigated on the calcium channel blocker amlodipine as a model drug compound. It was found that the specific biomimetic efficiency of nanofibers with metalloporphyrin-magnetic nanoparticles (FeTPPS_4_-MNP/PLA) is remarkably enhanced, compared to the same system FeTPPS_4_-MNP/PLA as a thin film form or to simple MNP bound FeTPPS_4_ systems [36].

### 3.4 Sensors

Electrospun NFs have been studied for the development of electrochemical sensors due to their high porosity, (gas) permeability, and high surface area with large number of active sites for enhanced sensing. Güzel et al., developed a caffeine-sensitive electrospun polyacrylonitrile-(5,10,15,20-tetrakis(4-*terc*-butylphenyl)porphyrin)/carbon felt electrode (PAN-^t^BTPP/CFE) nanofiber electrochemical sensor [37]. It was found that the ^t^BTPP was assembled into PAN nanofibers via π-π stacking, and this provided accelerated electron transfer in electrochemical caffeine oxidation.

In addition, electrochemical sensors, and optical sensors for the detection of various harmful gases have been developed using electrospun fibers immobilized with porphyrins. Generally, optical sensors are intrinsically safe in explosive environments and harsh conditions due to their non-electric nature and provide remote real-time monitoring. Mesin and Chu developed a sensitive and selective optical dual sensor for detecting NO and O_2_ simultaneously [38]. The sensor is based on the fluorescent complex 5,10,15,20-tetrakis(pentafluorophenyl)porphyrinato]platinium(II) (PtTPPF_20_) immobilized within cellulose acetate (CA) electrospun fibers for O_2_ sensing. Then, the fibers were also coated with CsPbBr_3_ perovskite quantum dots (PQDs) for NO sensing.

Moreover, core-shell electrospun nanofibers doped with fluorescent porphyrin dyes have also been used for the development of optical dual sensors for the simultaneous detection of gases. Putro et al. [39], studied an optical dual sensor consisting of a core cellulose acetate (CA) fiber containing PtTPPF_20_ for oxygen (O_2_) gas sensing, and a shell also of CA nanofiber with immobilized Eosin-Y for ammonia (NH_3_) detection.

Thus, electrospun nanofibers combined with porphyrin derivatives can develop (dual) sensors with high cross-sensitivity, selectivity, recovery, and dynamic response. Furthermore, the core-shell fiber emerges as a promising approach for developing innovative optical sensors. It is to note that the formation of the electrospun core–shell fibers is affected primarily by the solution properties and the electrospinning process parameters. The solution parameters include mainly viscosity, and conductivity as well as concentration, solution-solvent miscibility and compatibility, and solvent vapor pressure [40]. Regarding process parameters, solution flow rate, applied voltage, tip-to-collector distance and nozzle geometry affect the formation and morphology of the core–shell fibers. The solution flow rate of the core is a critical factor as a high flow rate produces fibers with insufficient shell to immobilize the core compounds, while a low flow rate in the core produces fibers with inadequate core compounds.

In another study optical implantable oxygen sensors based on methacrylated [5,10,15,20-tetraphenylbenzoporphyrinato]palladium(II) (PdMATPPB) embedded in biodegradable electrospun polymer matrices (e.g. PCL, Gelatin, PLGA), for *in vivo* tissue oxygenation monitoring, were developed and evaluated under physiologically relevant conditions using a specialized fluorimeter. Operating within the tissue-optical window (λ_Ex_: 630 nm; λ_Em_: 810 nm), the sensors exhibited linear Stern-Volmer behavior (which is a desirable condition for reliable and predictable sensor performance), with phosphorescence decay curves providing insights into the chromophore environment. Single-component PCL sensors showed the highest sensitivity and mono-exponential decays, ideal for real-time monitoring. Blending PCL with gelatin reduced degradation time while preserving sensitivity and decay uniformity, and a core-shell PCL:gelatin-PCL composition further enhanced stability and degradation control. Conversely, PCL:PLGA blends reduced sensitivity and increased chromophore heterogeneity, making them less suitable for these applications. All sensors demonstrated stable Stern-Volmer plots even after accelerated hydrolytic aging, with PCL and PCL:gelatin systems showing the most promise for future biomedical applications due to their sensitivity, stability, and tunable degradation profiles [41].

The potential of PCL-porphyrin nanofibers for sensing O_2_ was also investigated in another study, where a dual optical sensing membrane for real-time monitoring of dissolved O_2_ (DO) and pH was developed. Herein electrospun PCL and cellulose acetate (CA) nanofibers incorporating TPPF_20_Pt were used for DO sensing (F1), while chitosan (CS) nanofibers coupled with fluorescein 5-isothiocyanate (FIuTC) were used for pH sensing (F2). The final dual sensor was built by electrospinning F2 on top of F1 [42]. This biocompatible sensing film effectively responded to a wide range of DO concentrations and physiological pH levels, making it ideal for monitoring extracellular acidification and O_2_ consumption in cells and bacteria. The film exhibits luminescent signal changes corresponding to variations in DO and pH, without interference between the two sensors, ensuring high sensitivity and stability.

Due to its excellent biocompatibility and chemical stability, the dual-functional sensor has strong potential for use in biotechnology research, as well as in environmental monitoring, biological manufacturing, and wound care, offering significant value for *in situ* dynamic monitoring across diverse fields.

A summary of the latest works on porphyrin-based electrospun nanofibers for life science applications can be found in Table 1, and other studies are summarized elsewhere [5,24].

**Table 1:** Overview of porphyrin-based electrospun nanofibers for life science applications.

Type of electrospinning	Electrospun Materials^a^	Porphyrins encapsulated^b^	Applications	Objective of the study	Main outcomes of the study	Ref.
Coaxial	PVA and Gel	TPPF_16_[S–CH_2_–COOH]_4_	Drug delivery for PDT	Localized delivery systems for the treatment of cervical cancer	Selective inhibition of cancer cell proliferation under light irradiation	[24]
Monoaxial	PLA	FeTPPS_4_	Drug metabolism	*In vitro* mimicking of hepatic drug metabolism	Biomimetic ability of nanofibers on the calcium channel blocker amlodipine	[36]
Monoaxial	EC	ZnTPPF_20_	Antibacterial nanofibers	PS to induce light-mediated antibacterial activity	*E. coli* inactivation	[19]
Monoaxial	g-PGA	TMPyP	Antibacterial nanofibers	PS to induce light-mediated antibacterial activity	*In vitro: S. aureus* and *E. Coli In vivo: S. aureus-*infected mice in wound model	[34]
Monoaxial	PAN	*^t^*BTPP	Electrochemical sensors	Caffeine detection	Enhanced sensing mediated by porphyrin accelerated-electron transfer in electrochemical caffeine oxidation	[37]
Coaxial	CA	[PtTPPF_20_) and CsPbBr_3_ perovskite QD	Optical (dual) sensors	Simultaneous detection of NO and O_2_	The sensitivities for NO and O_2_ sensing were 10.7 and 2.7, respectively	[39]
Coaxial	CA	Core: PtFPPF_20_ Shell: Eosin-Y	Optical (dual) sensors	Simultaneous detection of O_2_ and NH_3_	The sensitivities for O_2_ and NH_3_ sensing were 6.4 and 3.2, respectively	[43]
Monoaxial and Coaxial	PCL, PCL:Gel and PCL:PLGA	PdMATPPB	Sensors for O_2_	Characterize the O_2_ sensing capabilities of electrospun biodegradable sensor; 2) Investigate the effect of composition in the sensor properties	PCL sensors exhibited the highest sensitivity; gelatin blends decrease degradation time and keep monoexponential decays and high sensitivity; core-shell composition, PCL:gelatin-PCL, maintain desirable sensor characteristics	[44]
Monoaxial	PHB	Hemin	Medical applications: Antibacterial	Investigate hemin impact on the structure and properties of PHB materials.	Hemin significantly affects supramolecular structure, morphology and mechanical properties of PHB nanofibers. Hemin-PHB displayed antimicrobial properties	[[31], [32], [33]]
Monoaxial	PCL blended with CA	PtTFPPF_20_	Dual oxygen and pH detection sensors by including porphyrin into CA/PCL fibers	Develop a two layer sensor film: i) PtTFPPF_20_/ CA/PCL electrospun fibers for O_2_ detection; ii) electrospun chitosan/FITC fibers for pH detection	Electrospun sensing film shows good response to a wide range of dissolved oxygen concentrations and physiological pH during extracellular acidification, and oxygen consumption of cells and bacteria	[42]

a Poly(vinyl alcohol (PVA); gelatin (Gel); Ethyl-cellulose (EC); Poly(g-glutamic acid) (g-PGA); Polyacrylonitrile (PAN); Cellulose acetate (CA); Poly(ε-caprolactone) (PCL), Poly(D,L-lactide-*co*-glycolide); (PLGA); Poly(3-hydroxybutyrate) (PHB);

b see structures in Fig. 3B

## 4 Concluding remarks

Porphyrin-encapsulated materials hold significant promise in life sciences due to their unique properties and bioactivities. This literature survey highlights how porphyrins enhance the functionality of nanofibrous matrices in several applications, particularly biomedical applications, due to their unique photophysical, photochemical, and catalytic properties. Electrospinning is an effective technique for encapsulating porphyrins, allowing the production of nanofibrous materials with high surface area, controlled release, and enhanced functionalities. The combination of porphyrins' properties with electrospinning technology creates a platform for several potential innovative solutions within life sciences. Those include the development of new photodynamic approaches, and the creation of composite biomaterials that could be used for antimicrobial applications, biosensors, drug delivery, and drug metabolism. In addition, several studies presented in this paper also showed that porphyrins impact the morphology, nano-microstructure and the functionalities (e.g. mechanical, and antimicrobial properties) of the electrospun nanofibers, which is linked to the interactions between porphyrin and fiber material. Different studies also show that the use of core-shell electrospun fibers was beneficial to creating successful proof-of-concept porphyrin-encapsulated materials for life-sciences, highlighting the versatility of this technology.

Most of the research discussed has focused on readily available 5,1015,20-tetraarylporphyrin derivatives. To advance the field, future studies should incorporate synthetic reduced porphyrins, such as chlorins, bacteriochlorins and isobacteriochlorins, as well as natural derivatives like protoporphyrin-IX and chlorophyll analogs, into electrospun (nano)polymeric systems. These derivatives offer improved bioavailability, enhanced near-infrared absorption, and superior biodegradability, making them ideal for next-generation biomedical technologies.

Despite the promising studies, further research is needed in the field of electrospun nano-micro fibers containing porphyrin molecules. Such studies and scalability trials could play a pivotal role in advancing the applications of these materials from the research stage to innovative applied societal solutions.

## CRediT authorship contribution statement

**Ana C. Mendes:** Writing – review & editing, Writing – original draft, Resources, Formal analysis, Conceptualization. **Nuno M.M. Moura:** Writing – review & editing, Writing – original draft, Investigation. **M. Amparo F․ Faustino:** Writing – review & editing, Writing – original draft. **M. Graça P.M.S. Neves:** Writing – review & editing, Writing – original draft. **Ioannis S. Chronakis:** Writing – review & editing, Writing – original draft, Conceptualization.

## Declaration of competing interest

The authors declare that they have no known conflicting financial interests or personal relationships that could have appeared to influence the work reported in this paper.

## Data Availability

No data was used for the research described in the article.
